# Astrocyte senescence-like response related to peripheral nerve injury-induced neuropathic pain

**DOI:** 10.1186/s11658-023-00474-5

**Published:** 2023-08-15

**Authors:** Jingyi Du, Nan Cheng, Yifan Deng, Ping Xiang, Jianfen Liang, Zhenye Zhang, Ziqing Hei, Xiang Li

**Affiliations:** 1 Department of Anesthesiology, The Third Affiliated Hospital, Sun Yat-Sen University, Guangzhou, 510630 China; 2 Department of Medical Quality Management, Nanfang Hospital, Southern Medical University, Guangzhou, 510000 China

**Keywords:** Neuropathic pain, Peripheral nerve injury, Neuroinflammation, Senescence, Astrocyte

## Abstract

**Background:**

Peripheral nerve damage causes neuroinflammation, which plays a critical role in establishing and maintaining neuropathic pain (NeP). The mechanisms contributing to neuroinflammation remain poorly elucidated, and pharmacological strategies for NeP are limited. Thus, in this study, we planned to explore the possible link between astrocyte senescence and NeP disorders following chronic sciatic nerve injury.

**Methods:**

An NeP animal model was established by inducing chronic constrictive injury (CCI) to the sciatic nerve in adult rats. A senolytic drug combination of dasatinib and quercetin was gavaged daily from the first postoperative day until the end of the study. Paw mechanical withdrawal threshold (PMWT) and paw thermal withdrawal latency (PTWL) were evaluated to assess behaviors in response to pain in the experimental rats. Senescence-associated β-galactosidase staining, western blot analysis, and immunofluorescence were applied to examine the levels of proinflammatory factors and severity of the senescence-like response in the spinal cord. Lipopolysaccharide (LPS) was administered to induce senescence of spinal astrocytes in primary cultures in vitro, to explore the potential impacts of senescence on the secretion of proinflammatory factors. Furthermore, single-cell RNA sequencing (scRNA-seq) was conducted to identify senescence-related molecular responses in spinal astrocytes under neuropathic pain.

**Results:**

Following sciatic nerve CCI, rats exhibited reduced PMWT and PTWL, increased levels of spinal proinflammatory factors, and an enhanced degree of senescence in spinal astrocytes. Treatment with dasatinib and quercetin effectively attenuated spinal neuroinflammation and mitigated the hypersensitivities of the rats subjected to sciatic nerve CCI. Mechanistically, the dasatinib-quercetin combination reversed senescence in LPS-stimulated primary cultured astrocytes and decreased the levels of proinflammatory factors. The scRNA-seq data revealed four potential senescence-related genes in the spinal astrocyte population, and the expression of clusterin (CLU) protein was validated via in vitro experiments.

**Conclusion:**

The findings indicate the potential role of astrocyte senescence in neuroinflammation following peripheral nerve injury, and suggest that targeting *CLU* activation in astrocytes might provide a novel therapeutic strategy to treat NeP.

**Graphical abstract:**

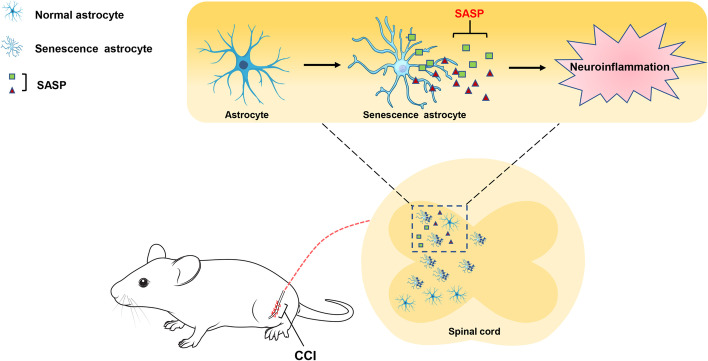

## Introduction

As a worldwide health issue, neuropathic pain (NeP) is reported to affect 3%–17% of the general population [1]. NeP is associated with sensory abnormalities and painful hypersensitivities, and clinically can lead to anxiodepressive consequences, thereby negatively impacting patients’ quality of life, which greatly exacerbates healthcare and social burdens and increases productivity losses [2, 3]. Although numerous efforts have been made to elucidate the mechanisms underlying NeP, therapeutic strategies for NeP remain limited [4]. There is increasing evidence indicating that some commonly used medications, such as antidepressants, nonsteroidal antiinflammatory drugs (NSAIDs), and opioids, might either provide limited analgesia or induce unpleasant side effects, including neurological disorders and opioid abuse [5]. Therefore, further investigation of the cellular and molecular aspects underlying NeP will aid in improving the choice of effective therapeutic agents to treat this neuropathic disorder.

It is well documented that recruitment of glial cells in the spinal cord following peripheral nerve damage leads to temporal over-production of inflammatory cytokines and chemokines, which may directly or indirectly contribute to neuroinflammation as well as central sensitization, and be ultimately implicated in the establishment and maintenance of NeP states [6, 7]. However, the mechanisms whereby glial cells release excess proinflammatory factors at the spinal cord level as a result of peripheral nerve injury are still unknown. Notably, the association between cellular senescence and neuroinflammation is subject to much debate. Senescence is described as a status of cell cycle arrest resulting from constant DNA injury or other stress-induced signals, such as telomere attrition, oncogenes, and cell stress [8]. Commonly, senescent cells exhibit a unique secretory phenotype, the senescence-associated secretory phenotype (SASP), which results in the generation of a wide variety of cytokines, chemokines, growth factors, and proteases [9]. For instance, expression of cytokines, such as interleukin (IL)-1β and IL-6, has been detected in senescent astrocytes in the aging rat brain, indicating that glial cells develop a proinflammatory phenotype in the senescent state [10].

Previous evidence from Shen et al. revealed an elevated intensity of senescence-associated β-galactosidase (SA-β-Gal) in sciatic nerve stumps of young adult rats (eight-week-old) that underwent sciatic nerve crush [11]. Additionally, Calls et al. reported that adult (20-week-old) mouse dorsal root ganglion sensory neurons expressed various senescence biomarkers including SA-β-Gal, phosphorylated H2AX, and nuclear factor kappa B (Nfkb)–p65 proteins, in a cisplatin-induced peripheral neuropathy model [12]. These studies suggest that the cellular senescence-like phenotype may not be in response to the aging process only, but also results from persistent nerve injury processes. Therefore, considering the potential roles of cellular senescence in the pathogenesis of neuroinflammation and peripheral nerve damage, we conducted the present study to elucidate the possible link between senescent phenotypes and NeP, and to identify whether inhibition of cellular senescence by senolytic agents could attenuate hypersensitivities resulting from chronic constrictive injury (CCI) of the sciatic nerve in adult rodents.

## Materials and methods

### Animals

Healthy male Sprague–Dawley rats (age 8 weeks, weight 220 ± 10 g) were obtained from Liaoning Changsheng Biotechnology Company (production license number: SCXK (Liao) 2020-0002, Benxi, Liaoning, China) without any pretreatments. All animal study protocols used were approved by the Animal Care Committee of the South China Agricultural University (approval no.: 2020d071), and strictly designed based on international laws and National Institutes of Health policies, including the Guide for the Care and Use of Laboratory Animals (8th ed., 2011). This study is reported in accordance with theAnimal Research: Reporting of In Vivo Experiments (ARRIVE) 2.0 guidelines [13].

All rats were reared in a specific-pathogen-free environment at the Laboratory Animal Center of South China Agricultural University, and housed in individual cages with a standardized circadian cycle (12–12 h day and night cycle), constant ambient temperature (24 ± 1 °C), and relative humidity (60% ± 5%). Chow and water were accessible ad libitum for all experimental rats.

### Pain model construction and drug treatment

**Fig. 1:**
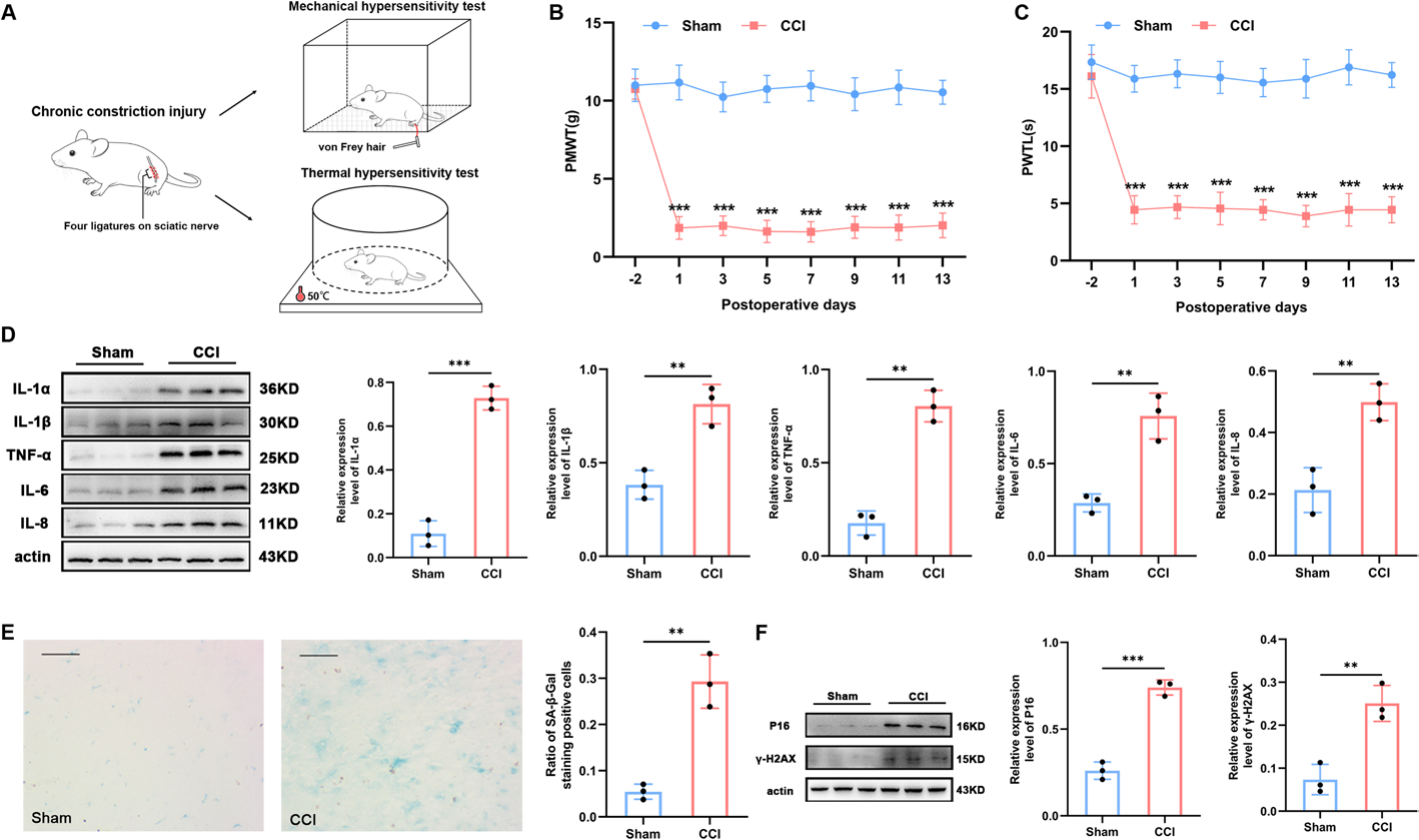
Rats experiencing sciatic nerve CCI exhibit mechanical and thermal hypersensitivities, inflammatory cytokine overproduction, and a cellular senescence-like response. **A** Experimental diagram illustrating sciatic nerve injury and behavior tests. **B**, **C** Mechanical allodynia and thermal hyperalgesia developed in the paws of the CCI rats and did not develop in the paws of the sham-operation rats from the first day after CCI until at least day 13 (*n* = 9). **D** Western blot images and quantification of *IL-1α*, *IL-1β*, *TNF-α*, *IL-6*, and *IL-8* in the spinal cord after sham intervention or sciatic nerve CCI in rats (*n* = 3). **E** Representative photographs of SA-β-gal staining (the positive cells show a blue precipitate) with statistical results (*n* = 3, scale bars 100 μm). **F** Western blot images and quantification for senescence-related markers *P16* and *γ-H2AX* in the spinal cord after sham operation or sciatic nerve CCI in rats (*n* = 3). **P < 0.01, ***P < 0.001

Based on a random number table, all 36 rats were randomly divided into the following four groups (*n* = 9 for each group): (a) sham, (b) CCI, (c) CCI with vehicle (CCI + veh), and (d) CCI with dasatinib and quercetin administration (CCI + DQ). The CCI rat model was established based on the methods proposed by Xie and Bennett to mimic the biology and physiology of NeP [14]. In short, with sufficient anesthesia depth induced with 2%–3% isoflurane (RWD Life Science Company, Shenzhen, Guangdong, China), the skin of the left hind limb of the rats was prepared, and a 1-cm skin incision was created below the femur. The subcutaneous tissue and biceps femoris muscle gap were bluntly separated to expose the sciatic nerve. The main trunk of the sciatic nerve was tied off four times using 4–0 chromic catgut with a spacing of 1.0–1.5 mm (Fig. 1A). The sham rats experienced identical treatment as CCI rats, except for ligation of the sciatic nerve. Postoperatively, all rats were fed a regular diet.

The dasatinib and quercetin combination is an effective senolytic drug combination that has been extensively used in previous preclinical studies [15, 16]. Therefore, for rats in the CCI + DQ group, 5 mg/kg dasatinib (SML2589, Sigma–Aldrich, Darmstadt, Germany) plus 50 mg/kg quercetin (Q4951, Sigma–Aldrich) were dissolved together in a solvent comprising 60% Phosal, 10% ethanol, and 30% polyethylene glycol-400, and gavaged daily, starting on the first postoperative day and continuing throughout the study. The above doses of dasatinib and quercetin were selected based on previous studies [17–19]. An equal volume of solvent was gavaged to rats in the CCI + Veh group over a comparable time period as that of the CCI rats receiving dasatinib and quercetin.

### Evaluation of pain-response behavior

As in our previous study [20], both mechanical and thermal sensitivities were measured to evaluate pain-response behaviors before and every other day after surgery (starting from the first postoperative day) by separately determining the paw mechanical withdrawal threshold (PMWT) in response to tactile stimulation or paw thermal withdrawal latency (PTWL) in response to heat stimulation (Fig. 1A). To enable the animal to adapt to the testing environment, all experimental rats were separately housed in a wire mesh grid (mechanical hypersensitivity test) or glass floored box (thermal hypersensitivity test) at least 1 h preceding behavior evaluation tests. For each rat, the PMWT evaluation was conducted at least 0.5 h before the PTWL evaluation.

The PMWT evaluation was performed using the von Frey hair test. In summary, a set of von Frey monofilaments (North Coast Medical, Morgan Hill, CA, USA) were used to perpendicularly stimulate the central plantar surface of the ipsilateral hind paw, starting from a stiffness force of 1.0 g. The filament test was repeated five times for each animal, with an interval of 5 s between each stimulation. The nociceptive behavior was regarded as paw withdrawal, accompanied by licking or shaking no less than three times over the course of five applications, and the minimum bending force of the monofilament required to initiate a nociceptive reflex was recorded as the PMWT value [20].

The PTWL evaluation was performed using a radiant heat apparatus (BME-480, Yuyan, Shanghai, China) that can continuously generate focused thermal stimulation onto the mid-plantar surface of the rat hind paw through the glass. To minimize the potential for cutaneous injury caused by heat stimulation to the rat paw, we selected a thermal intensity of 45 and a cut-off time of 25 s as previously described [21]. We repeated the stimulation five times with an interval of 10 min of rest for each rat. The average time between placement and first withdrawal sign from thermal stimuli (jumping and/or flinching) across the five tests was considered the PTWL value.

### Primary cell isolation, culture, and treatment

Astrocyte culture was conducted according to methods of previously published studies [22, 23]. Briefly, the spinal cords of newborn Sprague–Dawley rats within 3 days after birth (sex unidentified) were carefully isolated, and the dissected blocks of spinal cord tissue (1 mm^3^) were digested using 0.25% trypsin (40127ES60, Yeasen Biotechnology, Shanghai, China) at 37 °C for 15 min. DMEM/f12 medium (KGM12500N-500, KeyGEN BioTECH, Nanjing, Jiangsu, China) supplemented with 10% (v/v) fetal bovine serum (A3160802, Thermo Fisher Scientific Inc., Waltham, MA, USA) and streptomycin–penicillin solution (15140-122, Thermo Fisher Scientific Inc.) was added to end digestion. Subsequently, the cell suspension was harvested following centrifugation (800 rpm) for 5 min and incubated in 75-cm^2^ cell culture flasks (1 × 10^7^ cells/flask), which were placed in a 5% carbon dioxide cell incubator under a constant temperature of 37 °C. On the 12th day of culture, the culture flasks were shaken at 220 rpm for 10 h to obtain pure astrocytes. The floating cell suspension was discarded, and the remaining intact layer of astrocytes was trypsinized and subcultured. Immunofluorescence was used to confirm the identity of the cultured astrocytes using antibody against glial fibrillary acidic protein (GFAP) (16825-1-AP, Protentech Group, Rosemont, IL, USA), a specific astrocyte marker. Cultures with astrocyte purity exceeding 90% confirmed by *GFAP* immunoreactivity were used for subsequent experiments.

To induce senescence, the primary astrocytes were treated for 4 h with 200 μmol/L H_2_O_2_ (H_2_O_2_ group) or 1 μg/mL lipopolysaccharide (LPS, L4391-1MG, Sigma-Aldrich). Subsequently, 20 nmol/L dasatinib (SML2589, Sigma-Aldrich) plus 200 μmol/L quercetin (Q4951, Sigma-Aldrich) or DMSO was added to the LPS-induced astrocyte culture (LPS + DQ group, LPS + DMSO group) and incubated for 72 h. The Veh group was treated with DMSO. The LPS dose was based on the study by Nong et al. [24], and that of dasatinib plus quercetin was based on the work by Zhu et al. [15].

### Senescence-associated β-galactosidase staining

The SA-β-Gal staining procedure was carried out in line with the manufacturer’s protocols for the SA-β-Gal staining kit (C0602, Beyotime Biotechnology, Shanghai, China). Briefly, for cultured astrocyte staining, 1 mL SA-β-Gal staining fixative was added to each well and incubated for 15 min at room temperature. Subsequently, 1 mL β-galactosidase staining working solution was added to each well. The six-well plates were sealed with parafilm to prevent evaporation and incubated overnight at 37 °C.

For tissue staining, the L4–L6 spinal cord segment was dissected from rats on the 14th day after surgery and sectioned into 10-μm-thick cryostat sections using a freezing microtome (HM550VP, MICROM, Walldorf, Germany). Immediately afterwards, the sections were fixed in the SA-β-Gal fixative solution for 20 min at room temperature and then washed twice with phosphate buffered saline (PBS). After that, the sections were incubated overnight with the staining solution at 37 °C.

All SA-β-Gal staining images were observed under a light phase microscope (CKX41, Olympus, Tokyo, Japan). An investigator who had no knowledge of the group allocation details calculated the ratio of SA-β-Gal staining-positive cells via ImageJ software (version 1.8.0, National Institutes of Health, Bethesda, MD, USA).

### Western blot

According to previous studies, the spinal cord tissues at the L4–L6 level were harvested on postoperative day 14, and cultured astrocytes were lysed in radioimmunoprecipitation (RIPA) lysis buffer (KGP702, KeyGEN) supplemented with protease and phosphatase inhibitors. The total protein was collected by two rounds of centrifugation at 12,500 rpm at 4 °C for 15 min for each round. The BCA Protein Assay Kit purchased from Thermo Fisher Scientific (BCA-23225) was applied to determine the extracted protein concentrations. The proteins were separated using 10% SDS-PAGE gel electrophoresis and then transferred to PVDF membranes (ISEQ00010, Millipore, Darmstadt, Germany) using an electrophoresis technique. The membranes were blocked with 5% skim milk powder (BD 232100, Fisher Scientific, Pittsburgh, PA, USA) in Tris-buffered saline containing 0.1% Tween 20 (KGT539, KeyGEN) at room temperature for 60 min. The following primary antibodies were used for incubation: P16 (1:1000, 380928, ZEN-BIOSCIENCE, Chengdu, Sichuan, China), γ-H2AX (1:1000, 9718S, Cell Signaling Technology, Danvers, MA, USA), IL-1α (1:1000, A2170, ABclonal, Wuhan, Hubei, China), IL-1β (1:1000, A11369, ABclonal), TNF-α (1:1000, A0277, ABclonal), IL-6 (1:1000, A0286, ABclonal), IL-8 (1:1000, A2541, ABclonal), mucosa-associated lymphoid tissue 1 (MALT1, 1:1000, #2494, Cell Signaling Technology), enolase-1 (ENO1, 1:1000, A16841, ABclonal), clusterin (CLU, 1:1000, A12913, ABclonal), lactate dehydrogenase B (LDHB, 1:1000, A18096, ABclonal), actin (1:50,000, AC026, ABclonal), and tubulin (1:5000, AC008, ABclonal). After incubation with the primary antibody overnight at 4 °C, the membrane was washed and incubated with the secondary antibody (1:5000, 7074, Cell Signaling Technology) for 2 h at room temperature. We used the Tanon 5500 chemiluminescent imaging system (Tanon, Shanghai, China) to visualize the images, and ImagePro Plus (version 6.0, National Institutes of Health, Bethesda, MD, USA) to quantitatively evaluate the protein band intensities. As previously described [23], the relative expression level of the target protein was calculated by normalization to the intensity of the actin band.

### Immunofluorescence staining

Immunohistochemistry staining was carried out on the basis of our previous research [20]. For cell staining, the astrocytes cultured in 24-well plates were treated with 0.01% Triton X-100 solution for 20 min and blocked with 5% goat serum for 30 min. For tissue staining, after anesthesia with phenobarbital sodium (50 mg/kg) and intracardial perfusion with saline (100 mL) followed by 4% paraformaldehyde in PBS (60 mL), spinal cord enlargement tissues at the L4–L6 level were carefully collected from rats on postoperative day 14 and treated with 0.35% Triton X-100 solution followed by 5% BSA solution. A cryostat microtome (HM550VP, MICROM) was used to section the frozen specimens (thickness: 10 μm). The cultured astrocytes were incubated at room temperature overnight with the primary antibodies against *P16* (1:100, ab54210, Abcam, Boston, MA, USA), *IL-1α* (1:200, A2170, ABclonal), *IL-1β* (1:200, A11369, ABclonal), *TNF-α* (1:200, A0277, ABclonal),* IL-6* (1:200, A0286, ABclonal), and *IL-8* (1:200, A2541, ABclonal). The spinal tissues were incubated at room temperature overnight with the primary antibodies against *P16* (1:100, 380928, ZEN-BIOSCIENCE), *γ-H2AX* (1:200, 9718S, Cell Signaling Technology), *NeuN* (1:200, ab104224, Abcam), *Iba1* (1:150, ab15690, Abcam), and *GFAP* (1:300, 3670S, Cell Signaling Technology). Then, the cultured astrocytes and spinal tissues were incubated with Alexa 488-conjugated (1:500, ab150081, Abcam) or Alexa 594-conjugated (1:500, ab150120, Abcam) secondary antibody and treated with 4′,6-diamidino-2-phenylindole dihydrochloride (DAPI, G1012, Servicebio, Wuhan, Hubei, China) to mark the cellular nuclei. Photomicrographs were captured using the Thermo Fisher EVOS FL Auto laser confocal scanning microscope (Thermo Fisher Scientific Inc.). An investigator who had no knowledge of the group allocation details used ImageJ (version 1.8.0, National Institutes of Health, Bethesda, MD, USA) to calculate the ratio of overlapped area (senescence markers+/cell marker+) from the total cell marker-positive area for spinal tissue staining and the ratio of P16+/SASP+cell count to the total cell counts for primary astrocyte staining.

### Tissue dissociation and cell purification

In total, five rats (three for CCI and two for sham) were sacrificed on day 14 after surgery, and the spinal cord enlargement tissues at the L4–L6 level were dissected and immediately conserved in GEXSCOPE Tissue Preservation Solution (Singleron Biotechnologies, Nanjing, Jiangsu, China) with an ice pack for single-cell RNA sequencing (scRNA-seq). After washing thrice with Hanks’ Balanced Salt Solution (14025-076, Invitrogen, Waltham, MA, USA), the tissue was digested with 2 mL GEXSCOPE Tissue Dissociation Solution (Singleron Biotechnologies) at 37 °C for 15 min. Next, cells were filtered through 40-µm sterile strainers (CLS431750, Corning, Darmstadt, Germany) and centrifuged at 300*g* for 5 min. Then, the supernatant was removed, and the pellets were resuspended in 1 mL of PBS. To remove the red blood cells (RBCs), which were frequently a significant portion of the cells, 2 mL RBC lysis buffer (11814389001, Roche, Basel, Switzerland) was added to the cell suspension according to the manufacturer’s protocol. The cells were centrifuged at 500*g* for 5 min in a microfuge at 15–25 °C and resuspended in PBS. The sample from the cell mixture was stained with trypan blue (1450013, Bio-RAD, Hercules, CA, USA), and the cell concentration was adjusted to 1 × 10^5^ cells/mL. Subsequent sequencing analysis was performed when the cell viability exceeded 80%.

### Library preparation and sequencing

Single-cell suspensions (1 × 10^5^ cells/mL) in PBS were loaded into microfluidic devices using the Singleron Matrix^®^ Single Cell Processing System (Singleron, China). Subsequently, the scRNA-seq libraries were constructed according to the protocol of the GEXSCOPE^®^ Single Cell RNA Library Kits (Singleron Biotechnologies) [25]. Individual libraries were diluted to 4 nM and pooled for sequencing. Finally, pools were sequenced on Illumina NovaSeq 6000 (Illumina, San Diego, CA, USA) with 150-bp paired end reads.

### scRNA-seq quantification and analysis

Raw reads were processed to generate gene expression profiles using an internal pipeline. Briefly, cell barcodes and unique molecular identifiers (UMIs) were extracted after filtering read one without poly T tails. Adapters and poly A tails were trimmed before aligning read two to GRCh38 with ensemble version 92 gene annotation (fastp 2.5.3a and featureCounts 1.6.2) [26]. Reads with the same cell barcode, UMI, and gene were grouped together to calculate the number of UMIs of genes in each cell. The UMI count tables of each cellular barcode were employed for further analysis. Cell type identification and clustering analysis were performed by the R toolkit Seurat (version 3.0.1) [27]. UMI count tables were loaded into R using the read.table function. Afterwards, parameter resolution was set to 0.6 for the FindClusters function for clustering analyses. Differentially expressed genes (DEGs) between different samples or consecutive clusters were identified with the function FindMarkers with the thresholds of |log_2_FC|≥ 1 and adjusted *P* < 0.05. Gene Ontology (GO) function enrichment and Kyoto Encyclopedia of Genes and Genomes (KEGG) pathway analyses were carried out on the gene set using the R package clusterProfiler to explore biological functions or pathways that were significantly associated with the specifically expressed genes [28].

### Identification of differentially expressed senescence-related genes

The public Aging Atlas database (https://ngdc.cncb.ac.cn/aging/index) provides a platform for joint analysis of aging-related omics data, as well as online tools to visualize and compare these data [29]. In this study, we obtained 466 senescence-related genes (SRGs) from the Aging Atlas database. Thereafter, intersecting genes between DEGs and SRGs were acquired through a Venn diagram and defined as differentially expressed senescence-related genes (DE-SRGs).

### Statistical analyses

Data were expressed as means ± standard deviations (SDs), and all statistical analyses were performed with the SPSS software (version 20.0, IBM Corp., Armonk, NY, USA). The analyses for statistical significance of experimental data between two groups were carried out with the Wilcoxon–Mann *U* test (PMWT and PTWL), Student *t*-test (protein levels), and chi-squared test (ratios of SA-β-gal staining-positive cells and co-expression areas), while the data for more than two groups were analyzed using one-way or repeated measures two-way analysis of variance followed by Bonferroni post hoc comparisons. Statistical significance was set at *P* < 0.05.

## Results

### Rats undergoing CCI develop nociceptive behaviors and a senescence-like phenotype in the spinal cord

In contrast with the rats that underwent the sham intervention, those receiving sciatic nerve CCI displayed a significant progressive decrease in PMWT values when stimulated by von Frey monofilaments (*P* < 0.001 for each time point, Fig. 1B) and PTWL in response to a heat stimulus (*P* < 0.001 for each time point, Fig. 1C). Such tactile and thermal allodynia behaviors developed from the first day after operation and lasted for at least 14 days (Fig. 1B, C). In addition, increased neuroinflammation was exhibited in the form of enhanced levels of various proinflammatory cytokines (Fig. 1D), including *IL-1α* (*P* < 0.001), *IL-1β* (*P* = 0.005), *TNF-α* (*P* = 0.001), *IL-6* (*P* = 0.004), and* IL-8* (*P* = 0.006) in the spinal cord of CCI rats.

To prove the hypothesis that the senescence-like phenotype resulted from peripheral nerve injury, we investigated several molecular features of cell senescence in spinal cord tissues. First, spinal senescence was assessed using SA-β-Gal staining, a typical method for cellular senescence identification [30], and the proportion of SA-β-Gal-positive areas in CCI rats was considerably higher than that in sham rats (*P* = 0.002, Fig. 1E). Moreover, CCI-induced senescence in the spinal cord was also confirmed by increases in some molecular markers of senescence [31], such as *P16* (*P* < 0.001) and *γ-H2AX* expression (*P* = 0.001, Fig. 1F).

### CCI-induced cellular senescence mainly appeared in spinal astrocytes

**Fig. 2:**
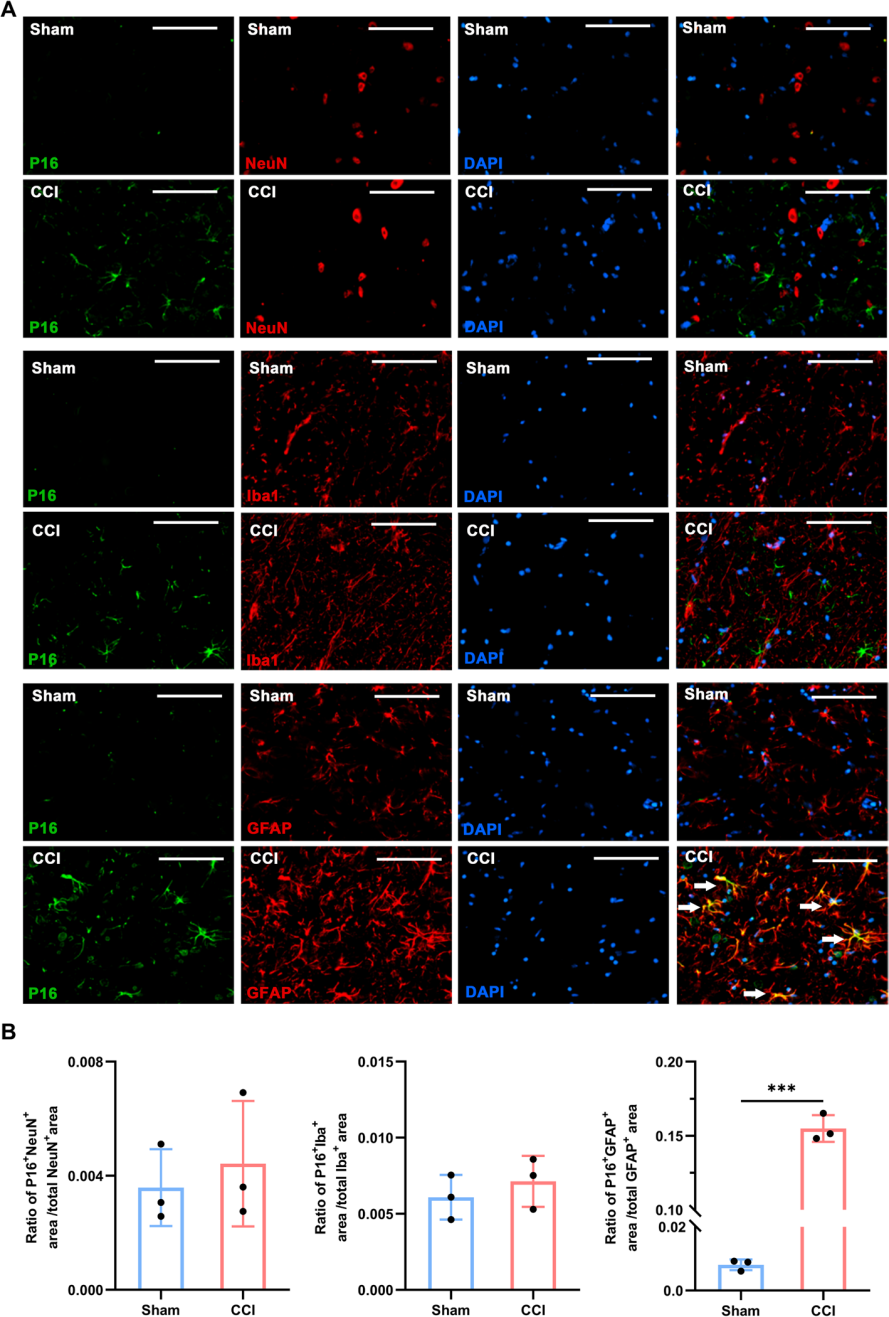
Double staining and quantification of *P16* in the spinal dorsal horn ipsilateral to sciatic nerve CCI or sham intervention. **A** Representative double-immunofluorescence staining images show the colocalization of P16 (green) with NeuN (neuronal marker)-, IBA1 (microglia marker)-, or *GFAP* (astrocyte marker)-positive cells (red). White arrows indicate colabelled cells. **B** Quantification analyses for the ratio of *P16* immunoreactivity area to neuron/astrocyte/microglia marker immunoreactivity area in the spinal cord (*n* = 3, scale bars 100 μm). ***P < 0.001

**Fig. 3:**
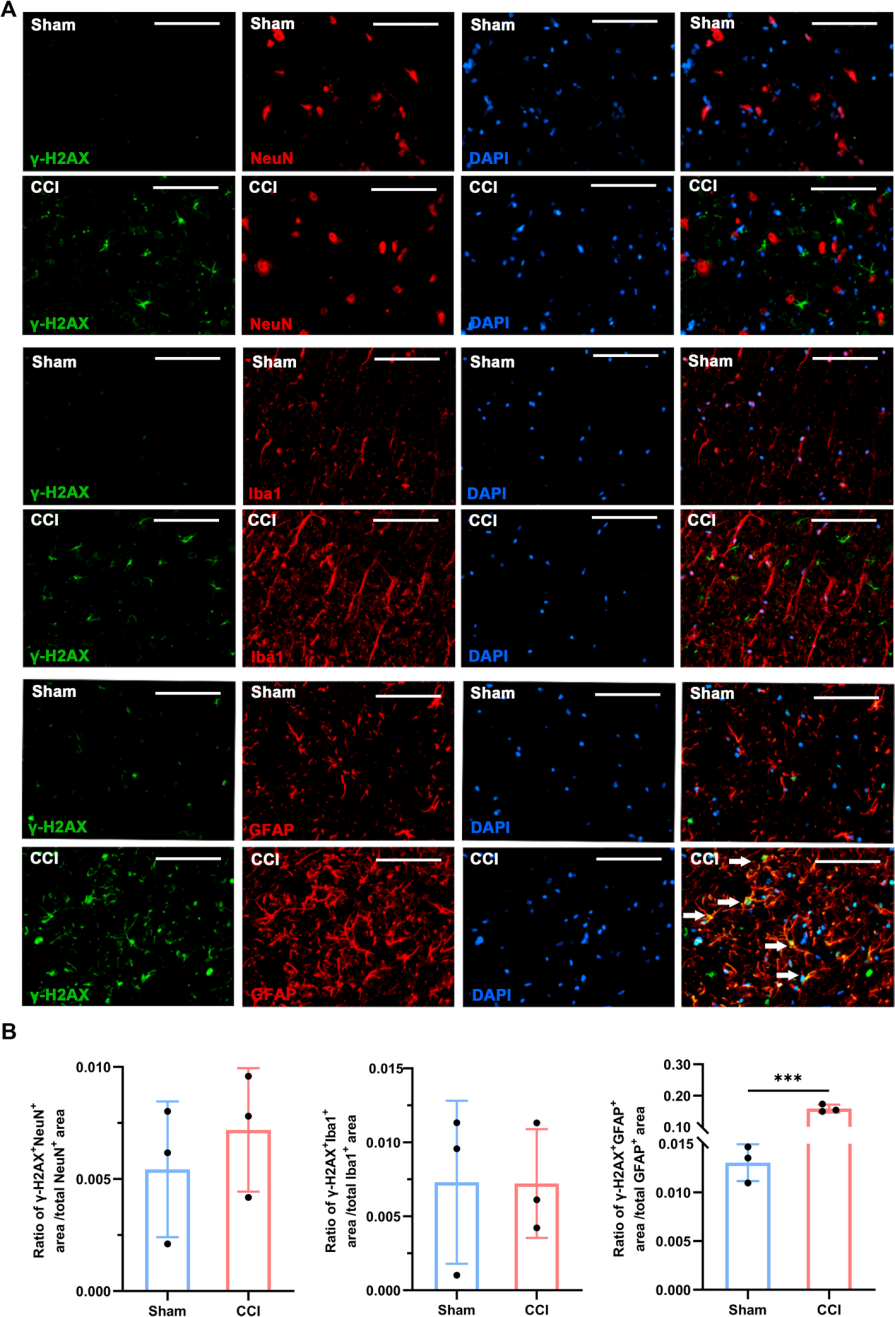
Double staining and quantification of *γ-H2AX* in the spinal dorsal horn ipsilateral to sciatic nerve CCI or sham intervention. **A** Representative double-immunofluorescence staining images show the colocalization of *γ-H2AX* (green) with NeuN (neuronal marker)-, *IBA1* (microglia marker)-, or *GFAP* (astrocyte marker)-positive cells (red). White arrows indicate colabelled cells. **B** Quantification analyses for the ratio of *γ-H2AX* immunoreactivity area to neuron/astrocyte/microglia marker immunoreactivity area in the spinal cord (*n* = 3, scale bars 100 μm). ***P < 0.001

To further determine the localization of senescent cells in the rat spinal cord following sciatic nerve CCI, double immunofluorescent staining was performed with three neurocyte markers. *P16*-positive (*P* < 0.001, Fig. 2) and *γ-H2AX*-positive (*P* < 0.001, Fig. 3) cells were costained with the astrocytic marker *GFAP* in the spinal cord sections following CCI surgery, whereas few *P16*- and *γ-H2AX*-positive cells were costained with the neuronal marker NeuN and the microglial marker *IBA1*. These results suggested that senescence resulting from peripheral nerve injury was likely to be predominantly exhibited in spinal astrocytes.

### Astrocytic senescence promotes the secretion of proinflammatory cytokines

**Fig. 4:**
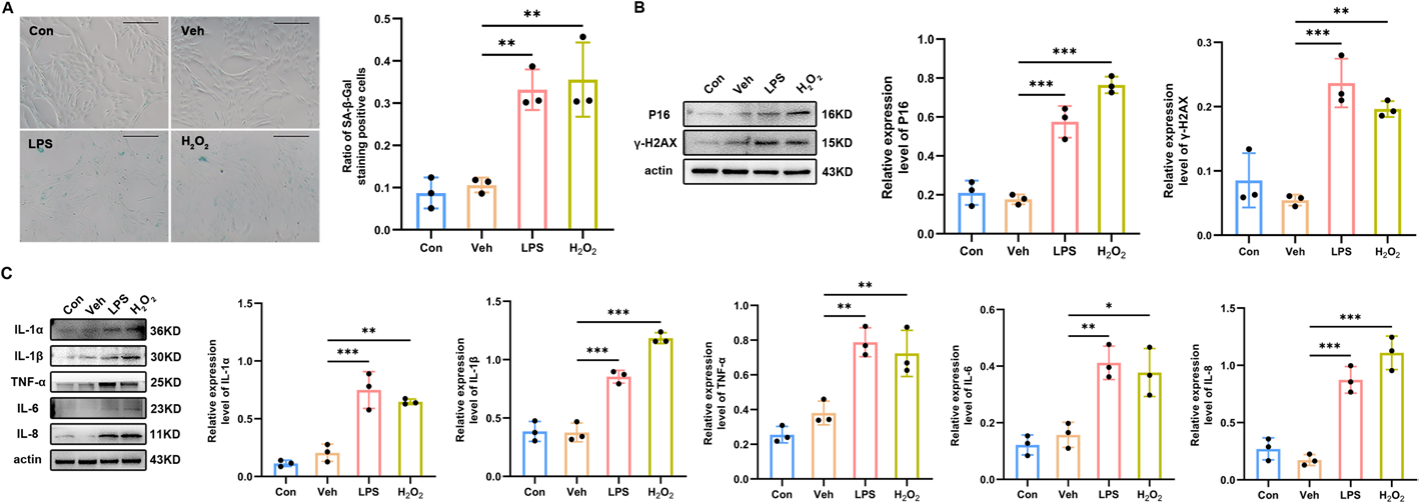
H_2_O_2_ and LPS induce senescence and inflammatory cytokine overproduction in primary cultures of spinal astrocytes. **A** Representative photographs of SA-β-gal staining (the positive cells contain a blue precipitate) and statistical results (*n* = 3, scale bars 200 μm). **B** Western blot images and quantification for senescence-related markers *P16* and *γ-H2AX* in primary cultures of spinal astrocytes (*n* = 3). **C** Western blot images and quantification for *IL-1α*, *IL-1β*, *TNF-α*, *IL-6*, and *IL-8* in primary cultures of spinal astrocytes (*n* = 3). *P < 0.05, **P < 0.01, ***P < 0.001

Similar to previous reports [32, 33], we used LPS to establish the in vitro neuropathic pain condition, and compared the effect of LPS on astrocytes with the effect of H_2_O_2_, a widely used inducer of cell senescence [34]. In the astrocyte cultures, the proportion of cells that robustly expressed SA β-Gal was increased after treatment with either LPS (*P*_Veh versus LPS_ = 0.005) or H_2_O_2_ (*P*_Veh versus H2O2_ = 0.003), when compared with the control (Con) cells (Fig. 4A). Furthermore, the promotion effects of LPS on P16 and γ-H2AX expression levels were comparable to those of H_2_O_2_ (P16: *P*_Veh versus LPS_ < 0.001, *P*_Veh versus H2O2_ < 0.001; γ-H2AX: *P*_Veh versus LPS_ < 0.001, *P*_Veh versus H2O2_ = 0.002; Fig. 4B).

In addition, the levels of major factors that belong to the SASP were greatly upregulated in LPS- and H_2_O_2_-exposed astrocytes, as determined by western blotting (Fig. 4C). Significantly greater expression was found for *IL-1α* (*P*_Veh versus LPS_ < 0.001, *P*_Veh versus H2O2_ = 0.002), *IL-1β* (*P*_Veh versus LPS_ < 0.001, *P*_Veh versus H2O2_ < 0.001),* TNF-α* (*P*_Veh versus LPS_ = 0.003, *P*_Veh versus H2O2_ = 0.009), *IL-6* (*P*_Veh versus LPS_ = 0.004, *P*_Veh versus H2O2_ = 0.011), and* IL-8 *(*P*_Veh versus LPS_ < 0.001, *P*_Veh versus H2O2_ < 0.001). Altogether, these results suggested that, at least in vitro, LPS can induce the senescence phenotype in rat spinal astrocytes, which then exhibit the characteristics typical for the inflammatory SASP.

### Senolytic treatment inhibits astrocytic senescence and alleviates pain behaviors

**Fig. 5:**
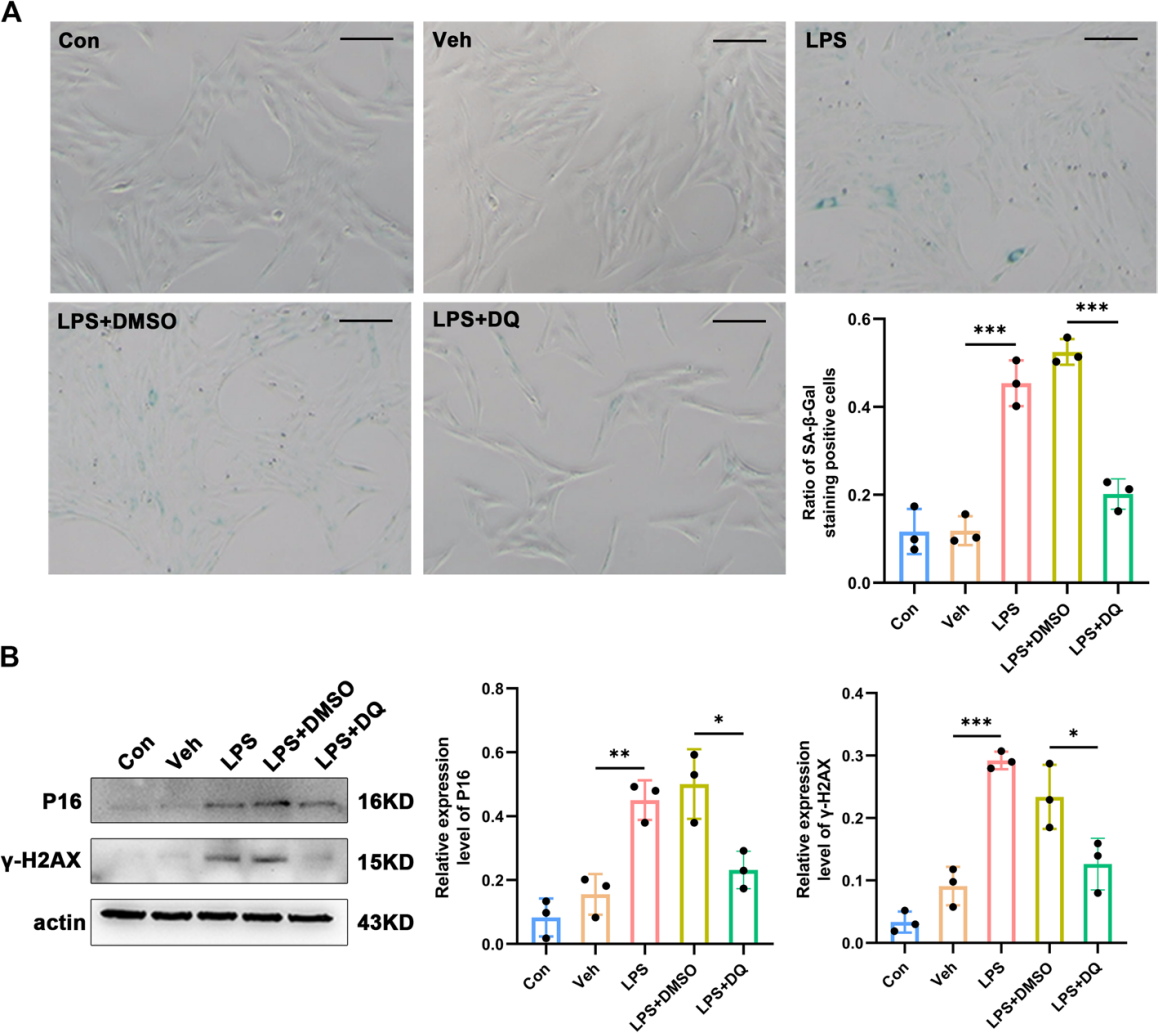
Dasatinib and quercetin delay senescence in primary cultures of spinal astrocytes. **A** Representative photographs of SA-β-gal staining (the positive cells contain a blue precipitate) and statistical results (*n* = 3, scale bars 100 μm). **B** Western blot images and quantification for senescence-related markers *P16* and *γ-H2AX* in primary cultures of spinal astrocytes (*n* = 3). *P < 0.05, **P < 0.01, ***P < 0.001

**Fig. 6:**
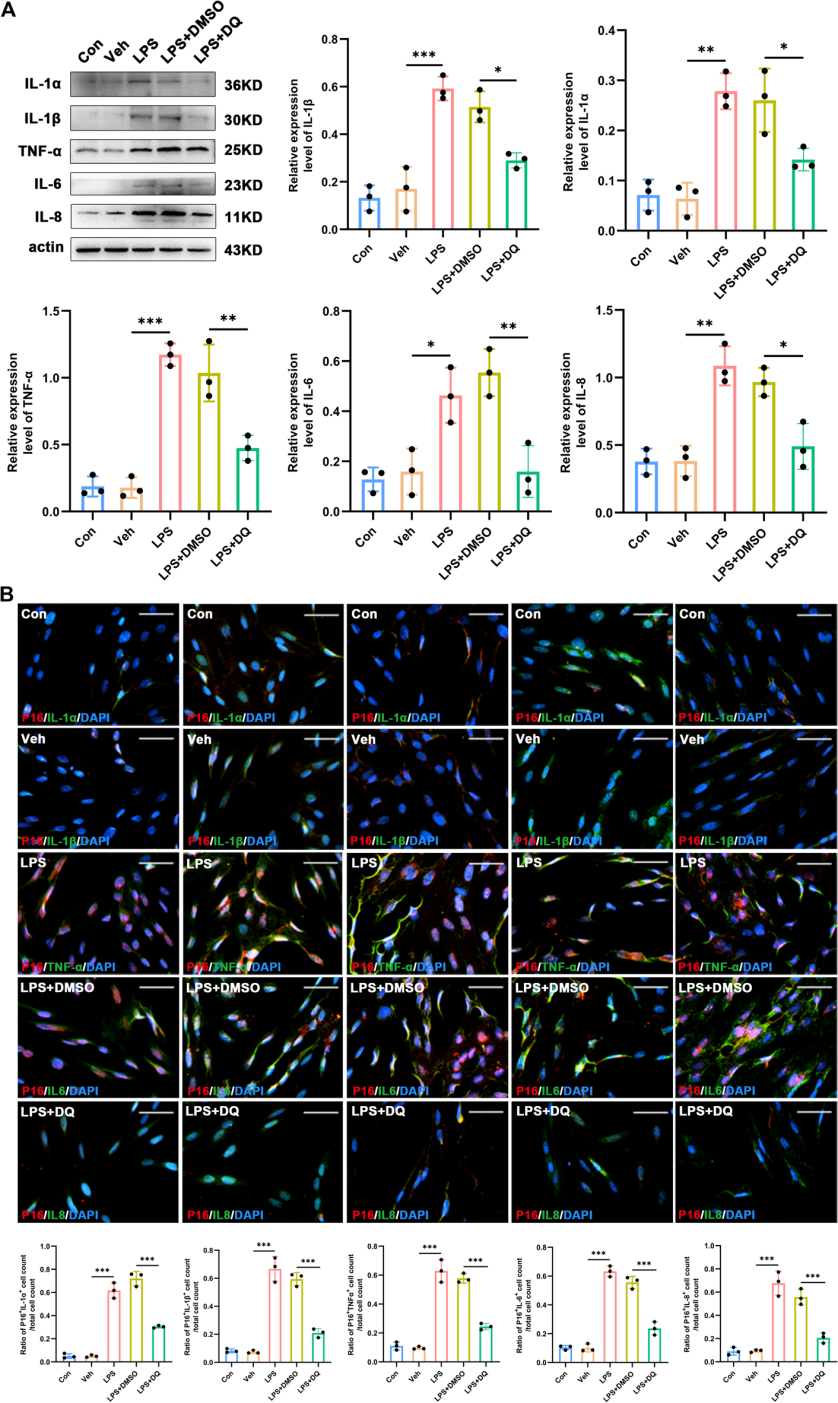
Dasatinib and quercetin reduce levels of inflammatory cytokines in senescent spinal astrocytes in vitro. **A** Western blot images and quantification of *IL-1α*, *IL-1β*, *TNF-α*, *IL-6*, and *IL-8* in primary cultures of spinal astrocytes (*n* = 3). **B** Representative images of double staining and quantification for the coexpression of *P16 *(red) and inflammatory cytokines (green) in primary cultures of spinal astrocytes (*n* = 3, scale bars 100 μm). *P < 0.05, **P < 0.01, ***P < 0.001

After confirming the occurrence of senescence in spinal astrocytes in NeP, we subsequently investigated whether senescent astrocytes participated in neuropathic pain progression. First, we explored the effect of senolytic treatment on senescent astrocytes in vitro. The data from the SA-β-Gal staining assay revealed that the ratio of LPS-induced senescent astrocytes was significantly reduced with 72-h treatment of a combination of dasatinib and quercetin (*P*_LPS+DMSO versus LPS+DQ_ < 0.001, Fig. 5A). Moreover, treatment of astrocytes with dasatinib and quercetin attenuated the elevations of *P16* (*P*_LPS+DMSO versus LPS+DQ_ = 0.011) and *γ-H2AX* (*P*_LPS+DMSO versus LPS+DQ_ = 0.031) expression levels in response to LPS (Fig. 5B) and reduced the critical SASP factors in senescent astrocytes, demonstrated by western blot and immunofluorescence staining (Fig. 6A, B).

**Fig. 7:**
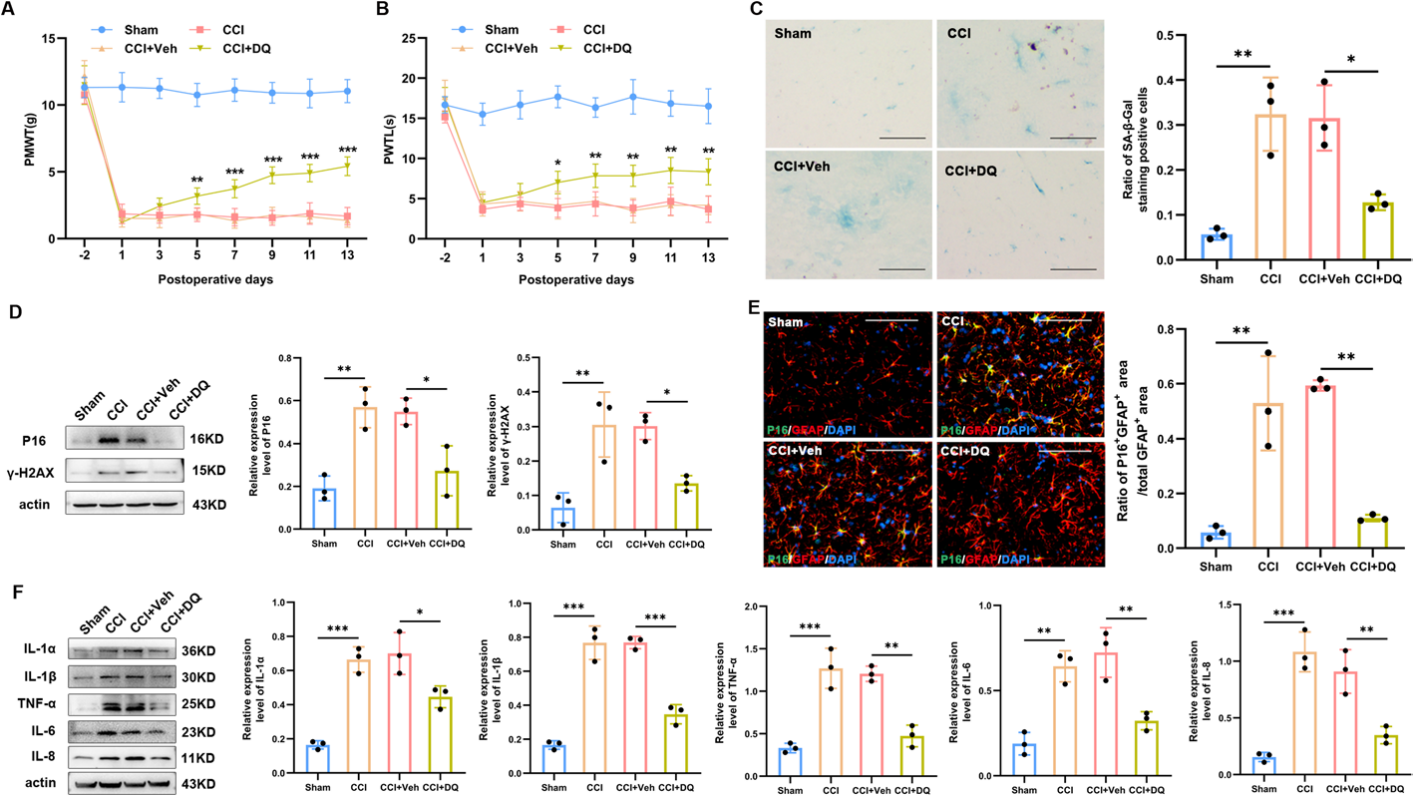
Dasatinib and quercetin inhibit senescence, alleviating CCI-induced pain, astrocyte senescence, and spinal cord inflammation. **A**, **B** Dasatinib (5 mg/kg) and quercetin (50 mg/kg) were gavaged to the CCI rats to detect mechanical allodynia and thermal hyperalgesia. The asterisks indicate the statistical significance of differences between the CCI + Veh and CCI + DQ groups (*n* = 9). **C** Representative photographs of SA-β-gal staining (the positive cells contain a blue precipitate) and statistical results (*n* = 3, scale bars 100 μm). **D** Western blot images and quantification for senescence-related markers *P16* and *γ-H2AX* in the spinal cord of rats (*n* = 3). **E** Representative images of double staining and quantification for the coexpression of *P16* (green) and *GFAP*, an astrocyte marker (red), in the spinal dorsal horn ipsilateral to sciatic nerve CCI or sham intervention (*n* = 3, scale bars 100 μm). **F** Western blot images and quantification of *IL-1α*, *IL-1β*, *TNF-α*, *IL-6*, and *IL-8* in the spinal cord of rats (*n* = 3). *P < 0.05, **P < 0.01, ***P < 0.001

In the in vivo study, we examined whether the senolytic agents could protect CCI rats against peripheral nerve injury-induced nociceptive hypersensitivity and astrocytic senescence. Compared with the vehicle treatment, oral administration of dasatinib and quercetin significantly improved the decrease in PMWT (*P*_CCI+Veh versus CCI+DQ_ = 0.009 at postoperative day 5, and *P* < 0.001 at postoperative days 7, 9, 11, and 13, Fig. 7A) and PWLT (*P*_CCI+Veh versus CCI+DQ_ = 0.016 at postoperative day 5, *P*_CCI+Veh versus CCI+DQ_ = 0.002 at postoperative day 7, *P*_CCI+Veh versus CCI+DQ_ = 0.001 at postoperative days 9, 11, and 13, Fig. 7B) in the CCI rats since the fifth postoperative day. Moreover, the CCI rats treated with dasatinib and quercetin showed a significant reduction in the ratio of SA-β-Gal-stained senescent cells (*P*_CCI+Veh versus CCI+DQ_ = 0.020, Fig. 7C) and expression levels of* P16* (*P*_CCI+Veh versus CCI+DQ_ = 0.026) and *γ-H2AX* (*P*_CCI+Veh versus CCI+DQ_ = 0.041, Fig. 7D). Immunofluorescence also demonstrated an increase in *P16* in the spinal astrocytes labeled by *GFAP* for CCI rats (*P*_Sham versus CCI_ = 0.001), which could be reversed by oral administration of dasatinib and quercetin (*P*_CCI+Veh versus CCI+DQ_ = 0.001, Fig. 7E). Western blot results indicated that senolytic treatment suppressed the secretion of SASP components in response to CCI (Fig. 7F), including* IL-1α* (*P*_CCI+Veh versus CCI+DQ_ = 0.027),* IL-1β* (*P*_CCI+Veh versus CCI+DQ_ < 0.001), *TNF-α* (*P*_CCI+Veh versus CCI+DQ_ = 0.001), *IL-6* (*P*_CCI+Veh versus CCI+DQ_ = 0.005), and *IL-8* (*P*_CCI+Veh versus CCI+DQ_ = 0.006).

### Identification of the intrinsic molecular response related to senescence in spinal astrocytes by single-cell RNA sequencing

**Fig. 8:**
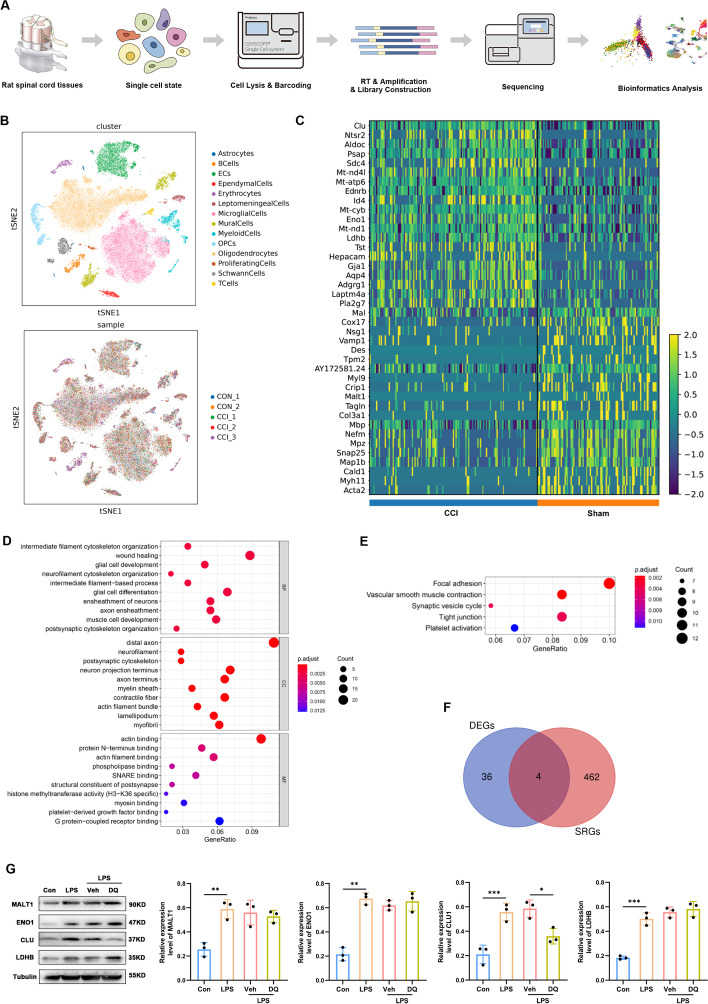
Single-cell RNA sequencing reveals profiling and transcriptional changes in rat spinal astrocytes in a neuropathic pain model. **A** Schematic overview of scRNA-seq analysis workflow. **B** t-distributed scholastic neighbor embedding (t-SNE) plot of single cells profiled in the present work and colored by cell types and samples. **C** Heatmap displaying the DEGs in spinal astrocytes from sham and CCI rats. **D** Bar plot displaying the significantly enriched GO terms for the DEGs in spinal astrocytes from rats following sham intervention or CCI. **E** Bar plot showing the significantly enriched KEGG pathways for the DEGs in spinal astrocytes from rats following sham intervention or CCI. **F** Venn diagram identifying the intersecting genes between DEGs and SRGs as DE-SRGs. **G** Western blot images and quantification of DE-SRGs (*MALT1*, *ENO1*, *CLU*, and *LDHB*) in the primary cultures of spinal astrocytes (*n* = 3). *P < 0.05, **P < 0.01, ***P < 0.001

The scRNA-seq was conducted as displayed in Fig. 8A. Visualization of scRNA-seq in the t-distributed stochastic neighbor embedding (t-SNE) space showed fourteen major cell types in the spinal cord, and the classical markers for astrocytes (*Aqp4*, *Ntsr2*, *Aldoc*, *Slc1a2*, *Agt*) corroborated the identification of this cell population (Fig. 8B). After identification of the astrocyte population, mRNA transcriptome profiles were compared in 95 and 131 spinal astrocytes from rats that had undergone the sham intervention or sciatic nerve CCI, respectively, and 40 DEGs were detected between the two groups (Fig. 8C). The results of GO analysis revealed that the DEGs were mainly enriched in intermediate filament cytoskeleton organization in the biological process category, distal axon and neurofilament in the cellular component category, and actin binding and protein N-terminus binding in the molecular function category (Fig. 8D). KEGG pathway analysis suggested that these DEGs were involved in focal adhesion, vascular smooth muscle contraction, and synaptic vesicle cycle pathways (Fig. 8E).

### Validation of the protein levels of potential senescence-related genes

After observing the intersection of DEGs and SRGs, *MALT1*, *ENO1*, *CLU*, and *LDHB* were defined as DE-SRGs (Fig. 8F). In the present in vitro study, the protein levels of four DE-SRGs were upregulated in response to LPS stimulation (*MALT1*: *P*_Con versus LPS_ = 0.004, *ENO1*: *P*_Con versus LPS_ < 0.001,* CLU:* *P*_Con versus LPS_ = 0.002,* LDHB:* *P*_Con versus LPS_ < 0.001, Fig. 8F), but significant reduction was only observed for the *CLU* protein level after dasatinib and quercetin treatment (*P*_LPS-Veh versus LPS-DQ_ = 0.026, Fig. 8G), which suggested the activation of *CLU* in astrocytes might be associated with senescence.

## Discussion

**Fig. 9:**
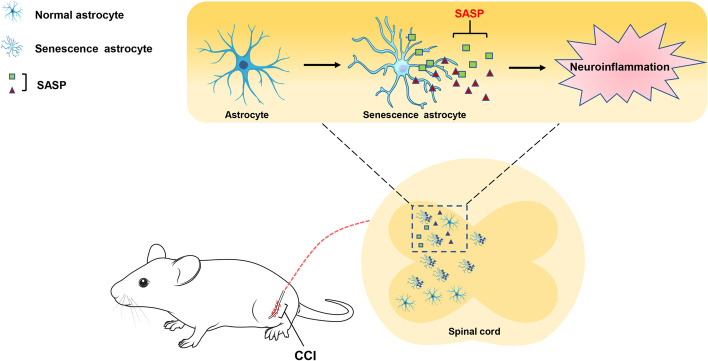
Mechanistic diagram illustrating the contribution of astrocyte senescence in neuroinflammation and neuropathic pain following peripheral nerve injury

Our findings suggest for the first time that astrocytic senescence may contribute to neuroinflammation at the spinal level and pain-like phenotypes resulting from sciatic nerve injury (Fig. 9). Prevention of astrocytic senescence by oral administration of a combination of senolytic agents (dasatinib and quercetin) attenuated spinal proinflammatory factor overproduction and hypersensitivities following CCI. These data indicate that senescence of spinal astrocytes possibly plays a critical role in the pathogenesis of NeP induced by nerve injury.

Accumulated evidence confirmed that the overproduction of proinflammatory cytokines and chemokines in the spinal cord is closely related to excessive activation of neuroinflammation following nerve injury and has some connection with the occurrence, as well as maintenance, of hypersensitivities [35, 36]. Consistent with these findings, the present study validated the occurrence of neuroinflammation following CCI surgery by revealing elevated levels of various proinflammatory cytokines in the spinal cord. Evidence has indicated the contribution of spinal astrocytes to the neuroinflammation in NeP caused by peripheral nerve injury [37, 38]. For instance, it was found that transgenic activation of the proinflammatory cytokine tumor necrosis factor (TNF) sourced from astrocytes aggravates mechanical hypersensitivity in a mouse neuropathy model [39]. Further, *IL-1β* from spinal astrocytes affect *IL-1* receptors and lead to enhanced neuronal and astrocytic activation, which ultimately extends the duration of neuropathic pain [40]. Because of such a close link between astrocytes and neuroinflammation in NeP, it is critical to consider how we can target astrogliopathy in the spinal cord.

Early studies have indicated astrocyte activation as the common feature of astrogliopathy in NeP; therefore, manipulation of astrocyte activity utilizing pharmacological treatments (e.g., astrocyte toxins or metabolic inhibitors that are preferentially taken up by astrocytes) was demonstrated to aid in alleviating neuropathic pain [37]. Remarkably, besides activation, our current study revealed for the first time that chronic peripheral nerve injury could also induce senescence in spinal astrocytes, which agreed with previous studies suggesting that astrocytes are capable of triggering the senescence phenotype in response to persistent nerve injury processes [11, 12]. More importantly, the SASP is considered a principal mechanism responsible for promoting inflammation by senescent cells, and it has been reported that senescent astrocytes in the brain display the characteristics typical of inflammatory SASP in a variety of aging-related pathologies, such as Alzheimer’s [41] and Parkinson’s [42] diseases. Our findings revealed that the secretion of inflammatory molecules, such as IL-6 and IL-8, was greatly stimulated in senescent spinal astrocytes treated with H_2_O_2_ or LPS in vitro. These findings provide some support for the conceptual premise that manipulating astrocyte senescence by senolytic agents could give researchers a new horizon for neuropathic pain management.

However, despite the causative relationship between senescence and neuroinflammation, the effects of senolytic treatment on preclinical models of NeP have not been investigated. Recently, the combination of dasatinib and quercetin has been used as a senolytic cocktail [17–19]. Dasatinib is a suppressor of small-molecule tyrosine kinase, which is capable of attenuating the viability of senescent cells, while quercetin could potently ameliorate oxidative stress injury and exert anticarcinogenic effects [18]. The senolytic cocktail comprising dasatinib and quercetin can selectively eliminate senescent cells without significantly impacting non-senescent cells, eventually reducing the count of senescent cells and secretion of proinflammatory SASP factors [43]. In the field of pain and analgesia, the combination of dasatinib and quercetin has been proven to be effective in alleviating painful phenotypes in various age-related pathological conditions, such as osteoarthritis [44] and intervertebral disc degeneration [18]. Intriguingly, the current study showed that the administration of dasatinib and quercetin in CCI rats significantly inhibited the overproduction of proinflammatory factors in the spinal cord and alleviated hypersensitivity behaviors. Based on the results of the in vitro study, which demonstrated the inhibition of LPS-induced senescence in cultured spinal astrocytes, we speculate that the combination of dasatinib and quercetin might act against peripheral nerve injury-induced NeP by eliminating senescent astrocytes and reducing SASP expression in the spinal cord.

In the field of neuropathic pain research, scRNA-seq is a powerful means for elucidating mutations at the level of individual cells [45]. In this study, enrichment analysis based on scRNA-seq of spinal astrocytes in a neuropathic pain model found the likely involvement of biological processes involving organization of the intermediate filament and neurofilament cytoskeleton. This finding is consistent with previous analyses, suggesting that the most common morphological alterations in senescent astrocytes are modifications in cell shape and intermediate filament reorganization [46, 47]. In addition, we screened four senescence-related genes (*MALT1*, *ENO1*, *CLU*, and *LDHB*) by comparing the lists of DEGs and SRGs, and validated the expression of *CLU* by western blot analysis. *CLU* is a conserved heterodimeric sulfated glycoprotein [48]. It was reported that *CLU *could regulate the expressions of SASP components (e.g., IL-6 and IL-8) through protein kinase B [49], Nfkb [50], or p38 mitogen-activated protein kinase (p38MAPK) pathways [51]. For example, Liang et al. demonstrated that the *CLU* could modify the secretion of SASP in hexavalent chromium-induced senescent L02 hepatocytes by regulating the *Nfkb* signaling pathway [50]. Indeed, we speculate that downregulation of *CLU* expression may inhibit nerve injury-induced astrocyte senescence and SASP secretion, and contribute to better treatments for NeP.

## Conclusion

Our data demonstrate a potential link between astrocyte senescence and spinal neuroinflammation in NeP. Elimination of senescent astrocytes by dasatinib and quercetin can effectively suppress the levels of proinflammatory factors in the spinal cord and reduce pain-sensitivity behaviors in rats. Among the four DE-SRGs identified by scRNA-seq, *CLU* has the potential to become a target for the development of drugs to inhibit astrocyte senescence in neuroinflammation following peripheral nerve injury. These results indicate that targeting astrocyte senescence might provide a novel therapeutic approach to treating NeP resulting from nerve injury.

## Data Availability

The data that support the findings of this study are available upon request from the corresponding author.

## Abbreviations

CCI: Chronic constrictive injury
CLU: Clusterin
DEG: Differentially expressed gene
DE-SRG: Differentially expressed senescence-related gene
DRG: Dorsal root ganglion
ENO1: Enolase-1
GFAP: Glial fibrillary acidic protein
GO: Gene ontology
IL: Interleukin
KEGG: Kyoto encyclopedia of genes and genomes
LDHB: Lactate Dehydrogenase B
LPS: Lipopolysaccharide
MALT1: Mucosa-associated lymphoid tissue 1
NeP: Neuropathic pain
Nfkb: Nuclear factor kappa B
NSAIDs: Nonsteroidal antiinflammatory drugs
p38MAPK: P38 mitogen-activated protein kinase
PBS: Phosphate buffered saline
p-H2AX: Phosphorylation of H2A.X variant histone
PMWT: Paw mechanical withdrawal threshold
PTWL: Paw thermal withdrawal latency
RBC: Red blood cell
SA-β-Gal: Senescence-associated β-galactosidase
SASP: Senescent-associated secretory phenotype
scRNA-seq: Single-cell RNA sequencing
SD: Standard deviation
SD rat: Sprague–Dawley rat
SPF: Specific-pathogen-free
SRG: Senescence-related gene
TBS: Tris-based saline
TNF-α: Tumor necrosis factor α
